# The Forgotten Life of Annie Reay Barker, M.D

**DOI:** 10.1093/shm/hkaa039

**Published:** 2020-05-31

**Authors:** Sophie Almond

**Keywords:** women doctors, microhistory, chronic mania, Holloway Sanatorium, Annie Reay Barker

## Abstract

Annie Reay Barker (1851–1945) was a medical pioneer who was amongst the first women to qualify as a doctor in the late nineteenth century. Unlike other medical women of her time, Barker did not attract notable attention or publicity, therefore little has been written about her personal and professional life. Following a successful, yet tragically short-lived, career at the Birmingham and Midland Hospital for Women, Barker was committed to Holloway Sanatorium in Virginia Water with a diagnosis of ‘Chronic Mania’. Barker’s story sheds new light on the pressures placed on early women doctors to succeed, as well as the troubled internal dynamics of this pioneering group of women.

On 23 March 1896, 44-year-old Dr Annie Reay Barker (1851–1945) was brought to the Holloway Sanatorium in Virginia Water with a diagnosis of ‘Chronic Mania’. Barker had excelled in her studies at both the University of Edinburgh and the Universitié de Paris, and was the eighth woman to have her name entered on the British Medical Register. She made history as the first female doctor to be appointed to a senior hospital position at the Birmingham and Midland Hospital for Women (BMHW) in 1878 and in recognition of her achievements, and respected status within the profession, was chosen to give the inaugural address at the London School of Medicine for Women (LSMW) in 1881. Once a successful physician with a promising career ahead of her, Barker found herself in the unfamiliar role of sanatorium patient, stripped of the authority and purpose that had defined her for two decades. Barker’s quiet departure from public life seemingly went unnoticed by her former colleagues, and her death nearly 50 years later similarly went unremarked in the medical press.

Until recently, histories of women in medicine have primarily focused on the successes that defined the movement, and have failed to delve deeper into the ‘failures’ which undermine the prevailing narratives of heroic sisterhood and triumph over professional adversity.^1^ In Barker’s case, her mental illness led to her professional successes being almost entirely erased from the historical record.^2^ Forgotten stories such as Barker’s deserve to be told. They provide a unique perspective on the varying levels of difficulty experienced by women attempting to establish themselves within the medical profession during this period, and provide further insight into the inner workings of this pioneering group of women.

Having proven, in qualifying, that they were as educationally able as men, the first generation of women doctors were tasked with establishing themselves as competent, dignified and resilient medical practitioners. They were expected to prove the legitimacy of women’s place within the profession by succeeding in all of their endeavours, whilst remaining resolutely dignified in the face of continued opposition. They were expected to be feminine, whilst also being mentally and physically robust; public knowledge of any mental or physical weakness would have fuelled the arguments of those who questioned the female sex’s ability to withstand the rigour of professional work.

Any mistake or temporary lapse in judgement might jeopardise the opportunities of future medical women. As Garrett Anderson notes in the 1878 *Medical Student’s Guide*:

It is necessary … to recognise that the standard of professional attainment expected by women, will for some years be higher than that expected of the ordinary male practitioner. Women can less easily afford to be second-rate; their professional work will be more closely scrutinised; mistakes will ruin them more quickly than they will men.^3^

If women doctors failed in their mission to prove themselves worthy, the doors to universities and senior hospital appointments would remain closed for the female sex in the decades to come. Not every woman doctor was willing, or able, to fulfil the exacting expectations of their role. As Drachman notes, ‘the struggle to become a physician was simply their first battle in the more enduring struggle to *be one*’.^4^ Having failed to meet the expectations of her profession, the details of Barker’s tragically short-lived career have been forgotten in the passing of time. This article redresses the balance, giving the forgotten life of Annie Reay Barker, M.D., the attention it so richly deserves.

## Elizabeth Garrett Anderson and Sophia Jex-Blake

In order to better understand the troubled internal dynamics of the professional group that Barker became a part of, one must first outline the tensions which existed between the movement’s self-appointed leaders, Elizabeth Garrett Anderson (1836–1917) and Sophia Jex-Blake (1840–1912). Faced with what seemed like an impossible task in the early 1860s, Garrett Anderson decided upon a measured, and most importantly non-confrontational, approach to gaining supporters for her pursuit of medical education. Rather than aggressively canvassing institutions, she made the strategic decision to procure private instruction from individuals, using her connections and ‘feminine charm’ to steadily win their respect.^5^ In a letter to Elizabeth Blackwell in 1861, Garrett Anderson wrote: ‘each doctor is willing to help me privately and singly, but they are afraid to countenance the movement by helping me in their collective capacity. This will, however, come in time’.^6^ After 4 years of careful negotiation, Garrett Anderson finally achieved her aim, and was permitted to sit for the examination of the Worshipful Society of Apothecaries (WSA) in September 1865.^7^ Having passed, she became the second woman to have her name placed on the British Medical Register.^8^

Following Garrett Anderson’s success in 1865, the WSA closed its doors to any further women hoping to secure their medical licenses.^9^ No medical institution in the UK would admit women as students, thus Jex-Blake was forced to take a more direct approach. If she went to Europe to gain her M.D., she would be unable to become officially registered on her return to England, and risked ‘hold[ing] a position exactly analogous to that of the most ignorant quack or herbalist who might open a penny stall for the sale of worthless nostrums’.^10^ Jex-Blake’s only hope of becoming fully qualified was to gain admission to one of the 19 institutions in the UK which were recognised under the Medical Act (1858).^11^

Faced with limited options, Jex-Blake adopted a strategy centred upon confrontation, choosing to forgo the calculated diplomacy embodied by Garrett Anderson. Having first met at a medical lecture in October 1861, Garrett Anderson concluded that Jex-Blake had a ‘jarring personality, with a judgement and temper she could not bring herself to trust’.^12^ Garrett Anderson was firmly of the belief that if any meaningful change was going to take place within the medical profession, valuable time would need to be spent winning over both hearts and minds. Jex-Blake, on the other hand, felt that the question of women studying and qualifying in the UK needed to be agitated and that the time for direct action was now. Rather than sharing Garrett Anderson’s long-term goal of gradually ameliorating the medical profession’s opinion of women doctors, Jex-Blake instead chose to focus all of her attention on the short-term issue of opening up British universities to women. This strategy proved to be fundamentally flawed. Not only did it fail to consider the effects that such confrontational behaviour would have on the perception of the movement as a whole, it also delayed Jex-Blake’s qualification by a number of years.^13^

Antagonism thus festered at the heart of the woman-doctor movement. Garrett Anderson and Jex-Blake continued to disagree, both privately and publicly, on how progress within the medical profession should be achieved and what the professional identity of the woman doctor should be. These tensions had a marked effect on first-generation medical women, including Barker, who faced the impossible task of navigating these competing strategies for professional progress and recognition.

## The University of Edinburgh

Annie Reay Barker was born on 20 April 1851 in Stoke-on-Trent to Dr Edmund John Barker and Emma Rowland.^14^ Barker’s father was said to have owned a large and fashionable medical practice in North Hampshire, and was described as the most busy and well-liked practitioner in the local area.^15^ In spite of the sex of his first born, Dr Barker was determined that his daughter would receive an education. He sent her to study at the pioneering Ladies Collegiate School in Belfast for a number of years, under the tutelage of the revered educationalist Margaret Byers (1832–1912).^16^ Byers founded the school in 1859, and was known to encourage her students to strive for academic excellence alongside giving them lessons in conventionally feminine subjects such as home-making.^17^ Inspired by her father’s successful practice, her teacher’s progressive attitude towards the education of women, and the events which were beginning to unfold in Edinburgh, Barker resolved that she would pursue a medical career.

In order to gain as much public support as possible, Jex-Blake widely publicised her campaign to gain admission to the University of Edinburgh in the national press. This strategy was a calculated one. By drawing unwanted attention to the injustice of women being excluded from Edinburgh, Jex-Blake hoped to force the University’s hand; they could either allow women to matriculate or face serious reputational damage. After her victory against the Senate in July 1869, Jex-Blake requested in *The Times* that ‘it would be well if any ladies intending to join these classes would at once communicate with me on the subject’.^18^ Barker was finishing her final year of studies in Belfast, and was therefore resigned to watch the group’s initial progress from a distance. But she was evidently determined to follow in Jex-Blake’s footsteps, and join the women already established in Scotland. Aged just 19, Barker was considerably younger than Jex-Blake and her circle; on first matriculation in 1869 Jex-Blake herself was 29, Edith Pechey (1845–1908) was 24, Matilda Chaplin Ayrton (1846–83) was 26 and Helen Grant (1833–1903) and Isobel Thorne (1834–1910) were both 35.^19^ In spite of her youth, Barker entered the fray by matriculating in medicine during the summer session of 1871, alongside Anna Dahms (1848–1917) and Jane Russell Rorison (1847–1915).^20^

Jex-Blake, Pechey, Chaplin Ayrton, Grant, Thorne, Mary Anderson (1837–1910) and Emily Bovell (1841–85) have long been lauded as ‘The Edinburgh Seven’, but as Crowther and Dupree note, this designation is inherently problematic.^21^ Contrary to popular belief, 10, not 5, women signed the matriculation register alongside Jex-Blake for the winter session of 1869, and by 1873, 39 women had joined the Edinburgh queue.^22^ Having been rejected from Jex-Blake’s exclusive ‘Septem contra Edinum’, the names of Elizabette Ken (1842–?), Mary Cudell (1842–?), Emily Rosaline Masson (1835–1915), Mary Spalding Roberts Sinclair (1845–?) and Elizabeth Mary Clark (1842–?) have also been widely forgotten.^23^ These women were erased from the historical record because they failed to be mentioned in Jex-Blake’s *Medical Women* (1886), which provided, in her opinion, the only ‘complete and comprehensive’ first-hand account of the ‘Battle in Edinburgh’.^24^

Written 13 years after Jex-Blake’s defeat in Scotland, *Medical Women* (1886) sought to set the record straight: ‘My own opinion is that … there was no “failure”; I believe that it was the seed sown in tears in Edinburgh that was reaped in joy elsewhere.’^25^ Crowther and Dupree argue that it is difficult to see the Edinburgh medical women clearly because of the indignant attitude of Jex-Blake; passionate in her feminine friendships, she chose to erase from her account those who failed to meet her standards.^26^ Given the fact that Ken, Cudell, Masson, Roberts Sinclair and Clark all chose not to follow through on their intentions to receive a medical education after Edinburgh, it is perhaps unsurprising that they do not feature in *Medical Women* (1886). However, Barker, Dahms and Russell Rorison are similarly erased by Jex-Blake, in spite of the fact that all three women went on to become successful practitioners.^27^ Barker receives a single reference in Todd’s biography, as Jex-Blake proudly makes note in her diary that in the matriculation examinations, ‘Miss Barker’s Logic paper best ever had from medical students [*sic*]’, whilst ‘Miss Bovell’s French best in University except one Frenchman’s [*sic*]’.^28^ Evidently, Barker had made a good first impression; Jex-Blake was aware of her academic achievements, which served to strengthen the women’s case for inclusion in Edinburgh. Why, then, was Barker overlooked in Jex-Blake’s account of the victory won?

Crowther and Dupree suggest that Barker and Dahms were ostracised by Jex-Blake because neither stood alongside her in the legal challenge against the University of Edinburgh, as both had gone abroad to seek their medical degrees.^29^ This is not entirely accurate, as Barker, Dahms and Russell Rorison were all explicitly named as plaintiffs in the action brought against the university by Jex-Blake in June 1873.^30^ Their names were listed alongside three other medical students who were similarly missing from *Medical Women* (1886): Elizabeth Ireland Walker (?-?), Sophy Massingberd Mundy (1845-?) and Rose Ann Shedlock (1850–78).^31^ It is, however, likely that Jex-Blake would have viewed the departures of Barker and Dahms to Europe as betrayals; after the legal action against the University of Edinburgh failed, Jex-Blake refused to accept that the cause was lost. She publicly decried Garrett Anderson’s suggestion that ‘by going to Paris female students can get, *without further difficulty or contention* … a first-class medical education’, retorting that she was ‘thoroughly resolved to “fight it out on *this* line”’.^32^ Jex-Blake firmly believed that Garrett Anderson’s cautious pragmatism would do nothing to further the cause of medical women in the UK; by encouraging women to go to Europe, the ingrained inertia of the British medical establishment would remain unchallenged.

Another possible explanation for Barker’s exclusion from *Medical Women* (1886) is the fact that, by the date of its publication, it had been 3 years since she had left her position at the Birmingham and Midland Hospital for Women, and was only practising medicine intermittently in London. Unlike Dahms, who had a successful dispensary in Manchester, Barker had, on account of her illness, effectively retired from hospital practice at the age of just 35, thereby ensuring that her 2 years spent in Edinburgh would be deftly excised from the authoritative history of those tumultuous years.^33^

## The BMHW

Having completed her medical degree in Paris, Barker returned home to Aldershot in search of her first junior hospital position. Whilst the war was being fought in Edinburgh, quieter victories were being won elsewhere. In July 1872, Louisa Atkins (1842–1924) was controversially employed by the BMHW as the country’s first female House Surgeon.^34^ In contrast, the Royal Free Hospital (RFH), which exclusively provided training to female medical students from the LSMW, did not appoint a newly qualified woman doctor to a house post until 1901.^35^ Because Atkins’ diploma was awarded from the University of Zurich, it did not technically meet the specified requirements of the post.

Additionally, due to the fact that women were yet to be permitted to sit the examinations of any licensing bodies, Atkins was not fully registered. In spite of this, the hospital’s committee still offered her the position over two male candidates, stating that they had every reason to be satisfied with their decision.^36^ Three years later, Edith Pechey, one of Jex-Blake’s most admired Edinburgh comrades, succeeded Atkins at the BMHW.^37^ Her appointment was similarly controversial; yet to fully complete her medical degree, she was, for all intents and purposes, both unqualified and unlicensed. Once again, the BMHW chose to overlook this fact, arguing that Pechey’s certificates and testimonials ‘appeared to guarantee [her competence]’, and as such ‘they had no hesitation in appointing [her]’.^38^ Lockhart asserts that the Governors of the hospital kept abreast of national issues discussed in the medical press and were, therefore, sympathetic to the difficulties faced by women aspiring to be doctors.^39^ Arthur Chamberlain (1842–1913), the founder of the BMHW, was determined that his institution would be progressive from the outset.^40^ As a specialist voluntary hospital for women, there was significant scope for surgical innovation, especially within the growing field of abdominal surgery.^41^ Chamberlain hoped that the pioneering work carried out at the BMHW would be recognised across the country, and was adamant that neither prejudices nor natural aversion to change would hinder future progress.^42^ In light of the hospital’s dedication to medical advancement, it is perhaps unsurprising that the Governors of the BMHW were willing to take such a liberal view on the employment of women doctors.

Following in the footsteps of Atkins and Pechey, Barker responded to the advertised position of House Surgeon and Secretary at the BMHW in July 1876: ‘*Wanted … A Lady House Surgeon* … Candidates must have completed their medical curriculum, and must have obtained, or be in the position to obtain, their diplomas. Salary £50 per annum, with board’.^43^ Junior house posts such as this were highly sought after, as they represented the first rung of the hospital career ladder. Over the course of 2 years, newly qualified doctors would gain the clinical experience necessary to apply for more specialist roles.^44^ The advertised salary was just half that offered to men for comparable House Surgeon positions, suggesting that employing woman doctors had a persuasive financial incentive for the hospital.^45^ Having reviewed Barker’s application, the Medical Board made the unanimous recommendation to appoint her to the post:

The Hon Sec reported that an application from Miss A R Barker had been received in answer to the advertisement for candidates for the vacant post of House Surgeon and Secretary. Her certificates and testimonials had been received and submitted to the medical board from whom the following report letter had been received: ‘In the opinion of this board the certificates of Miss Annie Reay Barker, a candidate for the office of House Surgeon, are similar to those which were presented by Miss E Pechey for the same appointment’. … It was unanimously resolved that Miss A R Barker be elected to the post of House Surgeon and Secretary.^46^

Like Pechey, Barker had yet to complete her degree or attain a medical license. Once again, her academic record and personal references were accepted by the BMHW in their place. Her appointment, and subsequent hiatus to sit her final examinations in Paris, was noted in the hospital’s *Annual Report*:

Miss A R Barker[‘s] … manner of fulfilling the duties of her post are highly approved and appreciated by the Acting Staff. Miss Barker was for some time absent in Paris, on leave, for the purpose of submitting herself to the necessary examinations for the degree of Doctor of Medicine … She has now returned, and the Committee are happy to state she has been successful in her object.^47^

**Fig. 1:**
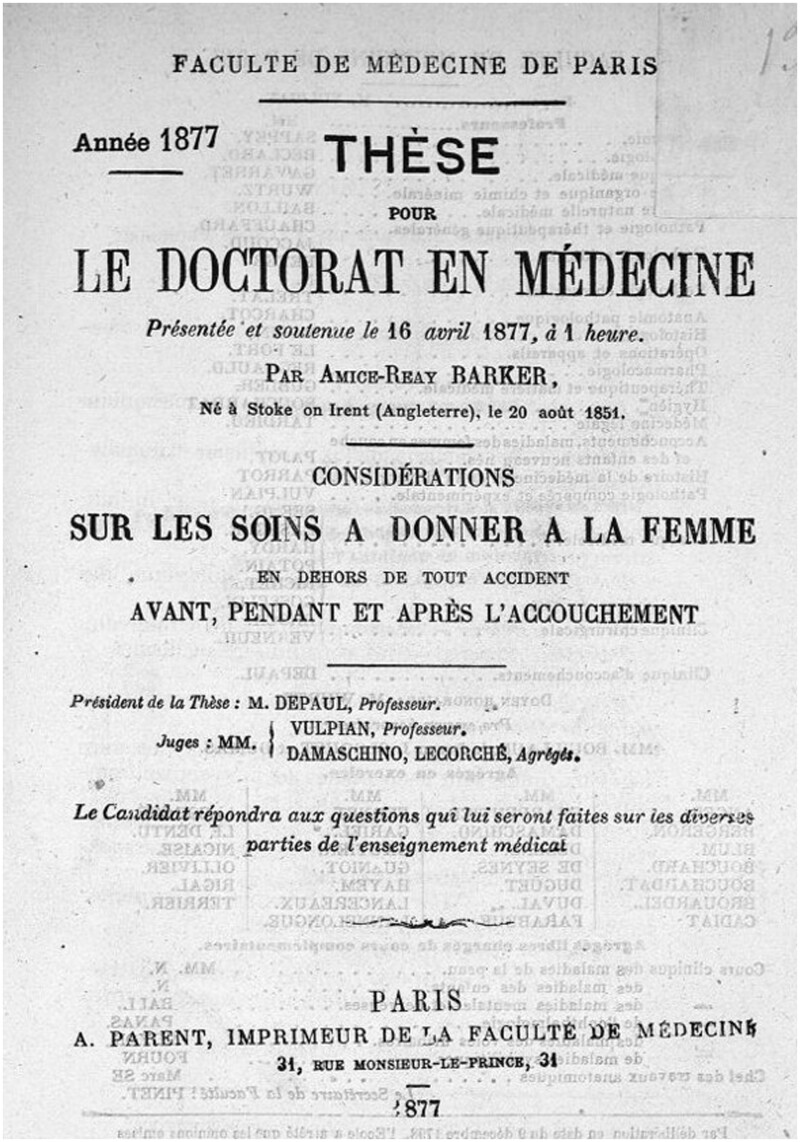
Barker's M.D Thesis (Image courtesy of BIU Santé, Paris).

In April 1877, Barker returned to Paris to defend her thesis: ‘Considérations sur les soins à donner à la femme en dehors de tout accident, avant, pendant et après l'accouchement’ (Figure 1).^48^ What had begun in Edinburgh 7 years previously was now complete. But Barker’s new title was not, in itself, enough to secure her right to practice medicine in the UK.

The Medical Act of 1858, which sought to standardise medical education, prevented those with European degrees from legally practicing in the UK. In 1876, the revised Medical Act was passed, which *enabled*, rather than *required*, licensing bodies to recognise ‘any qualification for registration granted by such body to all persons without distinction of sex’.^49^ The first medical body to permit women to take its licensing examinations was the Kings and Queens College of Physicians in Ireland (KQCPI).^50^ In January 1877, Eliza Walker Dunbar (1845–1924) became the first woman to be licensed by the College.^51^ The KQCPI played a fundamental role in the registration of women doctors; between 1877 and 1888, 48 medical women became licentiates.^52^ It is likely that the KQCPI’s generous concession was primarily motivated by the financial incentive of opening up their examinations to lady doctors. This being said, the College evidently sympathised with the difficulties that these women faced, knowing that by opening their own doors, other institutions would soon follow. Given that the KQCPI had already licensed Dunbar, Atkins, Pechey, Jex-Blake and Frances Hoggan (1843–1927), it would have been clear to Barker what her next step should be. In December 1877, Barker’s brother, Frederick, who was serving as a Red Cross Surgeon in the Russo-Turkish War, referred to her impending examinations in Dublin in a single surviving letter to his parents: ‘I wish Annie success and hope she will pass, that is the failure she speaks of Miss Jex Blake.’^53^ The failure referred to by Barker’s brother is unclear; after being awarded her degree from the University of Berne in January 1877, Jex-Blake passed the KQCPI licensing examinations in May of the same year.^54^

The KQCPI examinations were known to be rigorous, comprising a number of practical and written elements. Extracts from Jex-Blake’s diary, selectively quoted by Todd, convey her fear of failure on the eve of her departure to Ireland:

Off tonight for Dublin with E.P. Dr. A[tkins] also to join. ‘Omnne ignotum pro magnifico’. The various tests loom vague and large. Diagnosis at bedside,—horrible—though enormously helped by Brompton experience. Recognition of drugs and things under microscope. 4 written exams, 2 hrs. oral, etc., etc. I feel as if I really had fairly mastered my subjects and must know more than the average medical practitioner just fledged, - not to say have more sense. But the stake is so enormous. A pluck would be so perfectly awful after all antecedents [*sic*].^55^

As one of the primary figureheads in the campaign to open medical education to women, Jex-Blake was under an enormous amount of pressure to succeed in Ireland. In Edinburgh, this pressure had manifested in a very public ‘pluck’, when, much to the delight of her opponents, she had failed her examinations. In spite of her confident and uncompromising public façade, Jex-Blake was under acute psychological pressure to succeed in her endeavour to become fully-qualified. In a previous diary entry, written before the completion of her degree in Berne, Jex-Blake tellingly writes: ‘“If I fail it can’t be kept secret”. Are they all in league to shake my nerves?’.^56^ Failure would have meant public embarrassment; the small band of women vying to become doctors during the late-nineteenth century was in direct competition with one another, intimately aware of every success and failure. Fortunately, Barker’s hard work paid off, and she was spared the shame of falling at the final hurdle. On 9 January 1878, she signed the KQCPI’s roll of licentiates in medicine, becoming the eighth woman to have her name placed on the British Medical Register.^57^

Barker’s name has been most frequently associated with accounts of the BMHW’s progressive attitude towards women doctors; however, the historical significance of her time spent working at the hospital has repeatedly been overlooked. Being in full possession of her degree and medical license, Barker resigned from her junior position in April 1878.^58^ Fortuitously, shortly after Barker handed in her notice, two posts in the newly opened outpatient department at the BMHW were advertised—one for a Physician, and one for an Assistant Surgeon. The advertisement in the *Birmingham Daily Post* read as follows:

*Birmingham and Midland Hospital for Women* – There are *two vacancies* on the *Acting Medical* and *Surgical Staff*… Candidates must either be Fellows of the Royal College of Surgeons or have a Medical Degree, and be registered under the Act.^59^

Whilst the Act gave licensing bodies the authority to admit all candidates, regardless of sex, for examination, the Royal College of Surgeons refused to admit medical women as Fellows until 1911.^60^ The role of physician in the outpatient department, was, therefore, open to all qualified practitioners; however, no woman was qualified to apply for the surgical post.

Undeterred by this fact, Barker submitted her application along with two male candidates:

This meeting was called to examine and report upon the Diplomas, certificates, and testimonials of the candidates of the two offices of outpatient physician … Dr Annie Barker, Dr William Chubborn and Dr Robert Edginton each possessing a degree in medicine and being registered under the medical act, are qualified for a position upon the acting medical staff of the hospital.^61^

Lockhart incorrectly asserts that the two positions advertised were for a ‘Lady Physician’ and ‘Assistant Surgeon’, therefore inferring that Barker was not in direct competition with Chubborn and Edginton.^62^ The Board of Governors’ minute book clearly records the order of proceedings for the vote, which demonstrates, without any doubt, that Barker was on an equal footing to the male candidates:

Each voter is to be at liberty to give two votes, but only one vote to one candidate. A voter may, if he please, vote for one candidate only. The candidate having the greatest number of votes to be declared elected. A second ballot to be then proceeded with to decide between the two remaining candidates. In this vote each voter only has one vote. The candidate having the greater number to be declared elected’ … The result of the first ballot was as follows: Dr Annie Barker 31, Dr Chubborn 4, Dr Edginton 18. Dr Annie Barker was accordingly declared elected. In the second ballot Dr Chubborn received 5 votes, & Dr Edginton 28. Dr Edginton was declared elected.^63^

Barker’s decisive victory reflects the high regard in which her colleagues at the BMHW evidently held her; having been democratically elected to the hospital’s permanent staff, her character and professional expertise were shown to have taken precedence over her gender. Following her accession to the position of out-patient physician, the *Medical Examiner* was the only publication to note that: ‘the election [of Barker] is particularly interesting, as this is the first occasion on record in which a lady has, in competition with gentlemen, been chosen a member of honorary medical staff’.^64^

Having embarked upon her new role at the end of August 1878, Barker was immediately given the responsibility of sitting on the BMHW’s medical board alongside the eminent surgeon, Lawson Tait (1845–99). Tait was famed for his pioneering surgical techniques and was an outspoken supporter of female practitioners. Speaking publicly on the ‘woman question’ for the first time in 1874, he assured his colleagues that:

The number of women who would enter our profession would be a mere drop in the medical bucket, and as they would be the pick of their kind, they would undoubtedly be useful to us … it seems to me to be ridiculous, unwise, illiberal and impolitic to harbour a grievance by opposing them.^65^

Twelve years later, in his presidential address to the British Gynaecological Society, Tait reflected on his experience working with women doctors. Far from merely being ‘useful’ to medical men, they had surpassed all expectations by ‘harmon[iously]’ assimilating themselves into the working life of the hospital:

I have had a large personal experience of the medical education of women, and I have been intimately associated with a large number of women who have been trained and licensed as practitioners of medicine … In 1872 the Committee of the hospital to which I am attached, with a wise generosity which had then and has now my most complete approval, opened the appointments on the staff to women, and since then we have never been without a lady practitioner on our staff. We have had them as house surgeons, and we have had them as honorary medical officers, and nothing but the most perfect harmony has resulted from the combination … *there has not been the slightest attempt at friction of any kind*.^66^

For women doctors, their acceptance and successful integration within the hospital were reliant on their conformity to the traits, mannerisms and opinions of the male staff. As Garrett Anderson ironically put it, women doctors were expected to put aside their differences, and instead ‘peg away *manfully*’ at their work.^67^

One important aspect of the ‘perfect harmony’ which medical women brought to the BMHW was, according to Tait, protection from the ‘jealous eyes’ and ‘opposition’ faced by those who operated in the controversial department of gynaecology.^68^ Griffin argues that, in the political context, the collapse of Victorian domestic ideology forced male politicians to react defensively in order to protect both their positions of authority within society, and their sense of masculinity.^69^ Those who supported the women’s movement in parliament often had motives centred in protection and self-interest, rather than altruism.^70^ Similar defensive strategies can be found in the medical field. In a time when abdominal surgery involved such great risk, due to its experimental nature, the presence of ‘lady doctors’ on the hospital’s staff gave surgeons such as Tait increased security against reputational attacks:

In not a single instance was it ever possible for our critics to put their fingers upon an actually weak point in our proceedings … I attribute this to several reasons, but to no small extent do I attribute it to the fact that we have had a number of women on our staff … my lady doctors have ever proved on the one hand staunch and loyal colleagues, whilst their very presence has formed a *perfect bulwark of protection* against any charge our hostile critics hold against us. I do not think it would have been possible to have kept these women silent if it had been true, as was said of us, that we were performing unnecessary and improper operations upon their suffering sisters. It will be evident therefore that I have always felt a *sense of protection* in the fact of having a woman for a colleague.^71^

Throughout his career, Tait made great efforts to distance himself from the controversy attached to the ‘unnecessary and improper’ operations being performed on women, in spite of their similarity to his own surgical practice. Frampton notes that ‘Tait always denied using ovarian surgery to treat mental afflictions, but his operation [removal of ovaries and Fallopian tubes to cure inflammatory disease] was similar enough to Battey’s [removal of healthy ovaries to treat menstrual irregularities] that he repeatedly felt the need to emphasise their difference.’^72^ In response to the widespread vilification of gynaecological surgery, ovariotomists such as Lawson Tait rebranded themselves as ‘abdominal surgeons’, a term that, according to Frampton, ‘reflected the growing expansion of surgery’ whilst ‘allow[ing] practitioners to style themselves as unrestricted by gender … mean[ing] that their practice was less loaded with the risky sexual politics which specialists in female diseases frequently had to negotiate’.^73^ Barker’s appointment to the senior staff of the BMHW was, therefore, advantageous on a number of fronts. Not only did her expertise as a doctor benefit her own career, the fact that she was a woman also served the dual purpose of consolidating the hospital’s public image in an era of medical mistrust. Having women on the staff of the BMHW negated any accusations of ‘backwardness’ and instilled an atmosphere of progressive confidence, something which was crucial in an institution that exclusively dealt with the diseases of women.

## Association of Registered Medical Women

In spite of Barker’s success in Birmingham, the fight for professional recognition and equality for all women doctors was far from won. The British Medical Association (BMA) did not officially permit women doctors to join as members until 1892.^74^ Prior to this, Garrett Anderson was the only female member, having been discreetly accepted by the Paddington branch of the BMA in 1873.^75^ Once her membership became public knowledge 2 years later, a resolution was passed to prevent any further women from joining the Association.^76^ Medical women were, therefore, not only few in number, but they were also barred from joining the largest body of doctors in the UK. In May 1879, the Association of Registered Medical Women (ARMW) was formed in response to the professional isolation of women doctors.^77^ All 14 women on the medical register were invited to join the Association, but only 10 attended the ARMW’s second official meeting in 1880. In a further display of tension between early medical women, Hoggan, the first female doctor to receive a medical degree from a European university, refused to join the Association on account of the fact that women had to be fully registered in order to be eligible for membership.^78^ Those recorded as being present at the meeting were: Elizabeth Blackwell, Elizabeth Garrett Anderson, Sophia Jex-Blake, Louisa Atkins, Annie Reay Barker, Annie Clark (1844–1925), Mary Marshall (1837–1910), Matilda Chaplin-Ayrton, Eliza Macdonogh Frikart (1851–?) and Eliza Walker Dunbar.^79^

The Association held annual and later monthly meetings for its members, which typically included the sharing of papers, and the discussion of challenging cases. In later years, presidents of the ARMW would regularly write to the national newspapers, and the medical press, with their opinions on the pressing issues of the day, such as the medical education of women and women’s suffrage. Interestingly, the Association’s presidents were elected each year based on the order in which members’ names had been placed on the medical register. This practice supports the view that there was a well-established hierarchy among the medical women, with each being acutely aware of their precise position within it. Unsurprisingly, Garrett Anderson gave papers at meetings more routinely than any other member, and was elected president in 1881, 1896 and 1897.^80^ Whilst More claims that female medical societies functioned as ‘an effective instrument of professional integration and legitimation’, and were ‘agents of [both] feminism and professionalization [*sic*]’, they were also a form of professional control.^81^ Medical women who became fully registered during the year were ‘asked’, rather than ‘invited’, by the Secretary to join the Association before the next annual meeting, suggesting that there was an implicit expectation that every newly qualified medical woman should join the ARMW.^82^

**Fig. 2:**
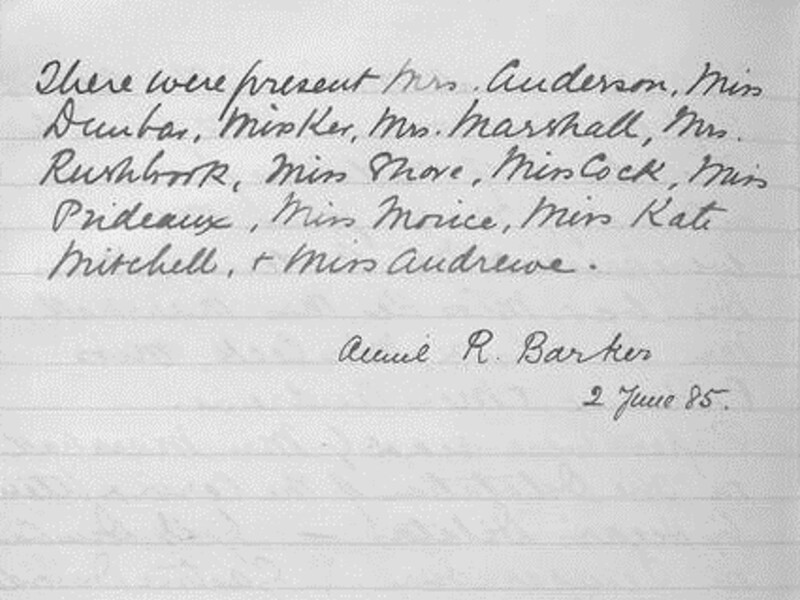
Barker's signature in the ARMW minute book (Image courtesy of the Wellcome Library, London).

The executive committee of the ARMW would have been anxious to attract as many members as possible in order to ensure their compliance with the ethics of the profession; as Edinburgh had shown, the actions of one medical woman had the propensity to affect the movement as a whole. In her brief discussion of the ARMW, Michaelson argues that: ‘The convivial atmosphere at branch meetings encouraged socializing [*sic*], thereby helping to foster an ethos of *sympathetic “sisterhood”* among medical women.’^83^ This analysis is flawed, as it fails to appreciate the intricate dynamics at play within this group of professional women. Beneath the façade of ‘sympathetic sisterhood’, women doctors were often at odds with one another, resistant to accepting the established hierarchy and the culture of conformity which was thrust upon them. In 1883, Jex-Blake sent a letter to the ARMW, proposing changes to the constitution.^84^ Evidently her suggestions were not addressed to her satisfaction, as the following year, she wrote again to withdraw her membership.^85^ Following Jex-Blake’s resignation, Barker was automatically appointed as President in 1885, and was later re-elected in 1888 (Figure 2).^86^

Whilst Barker had failed to win the esteem of Jex-Blake after their encounter in Edinburgh, her quietly committed work and unassuming nature did not go unnoticed by Garrett Anderson. In recognition of her position as a respected medical woman, Barker was afforded the honour of giving the inaugural address at the LSMW in October 1881. The founding of the school in 1874 had created further unrest between Garrett Anderson and Jex-Blake, as both held discordant views on the matter of co-education. Jex-Blake was enthusiastically in favour of a medical school for women, and was the primary figurehead behind the formation of the LSMW.^87^ After her experience in Edinburgh, she concluded that ‘boys of a low social class, of small mental calibre, and no moral training, are utterly unfit to be admitted to a mixed class’.^88^ Conversely, Garrett Anderson was initially reluctant to associate herself with the LSMW, as she believed that if co-education could be successfully established, medical women would be regarded as a permanent feature within the profession, rather than an experiment.^89^ Ironically, Jex-Blake left the LSMW after just 3 years, having been snubbed for the position of the school’s secretary.^90^ She later resigned from her position as trustee of the LSMW in 1897, following a disagreement over the school’s financial expenditure.^91^ Having thoroughly changed her view on the merits of a single-sex medical school for women, Garrett Anderson served as Dean of the LSMW for 20 years, and remained intimately associated with the school up until her death in 1917.^92^

Barker used her speech at the LSMW to publicly praise the BMHW for its progressive attitude towards employing women doctors, and, rather tellingly, urged the students sat before her to remain dignified in the face of adversity.

Miss Barker … gave a concise sketch of the history of the movement for the medical education of women, and then congratulated the students on the way in which they had worked to maintain its dignity and reputation … Miss Barker bore personal testimony to the progress which had been made in Birmingham, and expressed her pleasure in speaking of the fairness, practical good sense, and kind feeling with which medical women had been received there. The prejudices against women doctors must, Miss Barker told the students, be overcome, not by showing ill will in return, but by honest, true work, and by showing that, though they have entered a profession, they have lost none of the refinement and dignity of true gentlewomen.^93^

Barker’s address was noted to have attracted ‘a crowded meeting’, and was ‘received by all with much enthusiasm’.^94^ Having secured herself the medical education which she had fought so hard for, and being comfortably situated in a senior position on the acting staff of the BMHW, Barker had overcome the prejudices faced in becoming a woman doctor with her dignity and reputation firmly intact. Speaking during the time that, tragically, would later prove to be the climax of her professional life, Barker envisioned a future full of promise for herself, in the supportive environment of the BMHW, and for the students that had yet to embark on their own careers. What she did not yet know was that her career as an accomplished medical woman would shortly come to an abrupt end, and that she would fall victim to mental illness.

## Illness and Decline

Towards the end of 1882, Barker’s ill health began to affect her hospital work. Between August 1878 and August 1882, she had attended 38 out of 46 Medical Board meetings at the BMHW, sitting in the chair for eight of them.^95^ In September 1882, Barker was uncharacteristically absent from the monthly meeting, which marked the beginning of her decline: ‘A letter was received from Dr Annie Barker announcing that owing to ill health she was for the present absent from her duties on the outpatient staff.’^96^ Two months later, Barker noted a small improvement, but remained unable to perform her work at the hospital.^97^ Barker’s optimism for recovery was sadly short-lived; after 5 months leave, she was forced to write to the Medical Board expressing her regret that she could no longer continue in her position due to her ill health. Her resignation was accepted, and she was thanked by the Board of Governors for her services to the hospital.^98^ After Barker resigned from her post in Birmingham, she returned home to Aldershot. However, she continued intermittently to practice medicine privately in London. In 1885 and 1887, Barker’s address was listed in the *Englishwoman’s Review* as ‘37 Gloucester Place’, which was the same address used by Atkins between 1885 and 1889.^99^ Given Barker’s traceable activity in 1885, 1887 and 1888, it is likely that she experienced highs and lows in her health, which allowed her to continue working, albeit transiently, during this time.

Interestingly, Atkins had experienced similar periods of ill health, suffering at times from depression.^100^ In 1888, a heated professional disagreement between Atkins and Garrett Anderson led to Atkins resigning from her position on the staff of the New Hospital for Women (NHW), and as a member of the ARMW.^101^ Atkins had raised concerns regarding the surgical competency of Garrett Anderson, and when these concerns were not adequately addressed by the hospitals’ management committee, she saw no other option but to leave.^102^ Having hosted the annual meeting of the ARMW at her home in Hanworth in 1886 and 1887, it was ‘proposed and carried unanimously that next year’s meeting be held, subject to Mrs Atkins [*sic*] convenience, at the Rectory Cottage Hanwell, on the second Tuesday in June 1888’.^103^ Following Atkins’ resignation from the NHW in April 1888, the location of the annual meeting was, rather tellingly, changed to the ‘Inns of Court Hotel, Holborn’.^104^ The resignations of two founding members of the ARMW further demonstrates the discord that existed among the first generation of female medical graduates. As Elston notes, such disagreements between the early medical pioneers reveal a tension between the ideals of professional community, and individualistic conceptions of the role of women doctors.^105^ For many, Garrett Anderson’s elevated position within the profession was problematic; as the first woman to qualify in Britain, she was, in effect, irreproachable.

Following her resignation, Atkins continued to practice medicine privately in London and Northwood, but spent the rest of her life in solitude, distancing herself from her former friends and colleagues. Her death in November 1924 was described in her obituary as ‘characteristic; it was her wish that her death should not appear in the … papers … [this] was dictated by a proud humility, which valued itself at small price, and eschewed the ways of publicity’.^106^ In spite of her disagreement with Garrett Anderson, Atkins remained loyal to the ideals of professionalism which bound women doctors in solidarity with one another. Rather than publicly exposing the tension which had led her to resign from the NHW, Atkins instead removed herself from the London medical scene and quietly retired to the country.

The fact that both Atkins and Barker chose to live as recluses after leaving their respective hospital posts is telling. It suggests that there was an implicit expectation for medical women to remove themselves from the public eye when they were no longer capable of upholding the ideals of their professional identities. Failure would have been an ever-present fear; as a member of this exclusive band of women, defeat had the propensity to bring the whole group into disrepute, to undermine the sacrifices made by those who had come before and to fuel the prejudices of those who still believed that members of the female sex were incapable of being doctors. This relentless expectation to succeed similarly affected the second generation of medical women who qualified in the early twentieth century. In April 1902, Jeannie Macleod (1874–1902), a highly commended graduate from the University of Aberdeen, was found dead in the on-call room of Aberdeen Children’s Hospital, having severed her jugular.^107^ Macleod had only started working at the hospital a few days previously.^108^ Similarly, in August 1903, Sophia Frances Hickman (1874–1903), a prize-winning graduate of the LSMW, went missing from her post at the RFH, sparking a nation-wide search.^109^ Four weeks later, her severely decomposed body was discovered near Richmond Park.^110^ Post-mortem examination revealed that she had died from the effects of self-inflicted morphine-sulphate poisoning.^111^

Like Atkins, Barker’s withdrawal from public life was dictated by a ‘proud humility’ that eschewed any unnecessary publicity. In 1888, her residential address was once more listed as ‘The Mount, Aldershot’, which marked the end of her private practice in London.^112^ The following year, Barker was uncharacteristically absent from the annual meeting of the ARMW, having been an active member for over a decade. Barker’s resignation was not noted, as was usually the practice, in the Association’s minute book, which suggests that she did not write to her colleagues to inform them of her early retirement from the profession. Barker’s name continued to be listed on the LSMW’s Board of Governors until 1899, which further supports the view that she simply did not tell anyone that she was no longer practising medicine, and no one thought to ask.^113^ The reason why Barker avoided attracting any attention would have been due to the true nature of her illness. Mental instability attracted shame and embarrassment. Its causes were not fully understood, therefore those who suffered from diseases of the brain and disorders of the mind were treated as social outcasts, with their morality often being placed under question.^114^

As a woman, and a pioneering doctor, Barker’s illness would have been especially humiliating; she had devoted her professional life to proving wrong those who thought that women were incapable of dealing with the stresses of medical practice. Public knowledge of this incapacity would have tarnished Barker’s reputation, diminished the legacy of her career and undermined the medical women’s cause. As Brock notes, women doctors in this period were self-defined by a ‘robustness’ of both body and mind.^115^ This was a self-conscious characterisation motivated by the fact that public opinion—and large numbers of their male colleagues—continued to dispute women’s ‘mental, physical and moral capacity to act as members of the medical profession’.^116^ Sharing the full extent of her illness with her colleagues would have been an impossible task. It would have meant admitting weakness—something that had been stigmatised in the fight for women’s admission to the medical profession.

**Fig. 3:**
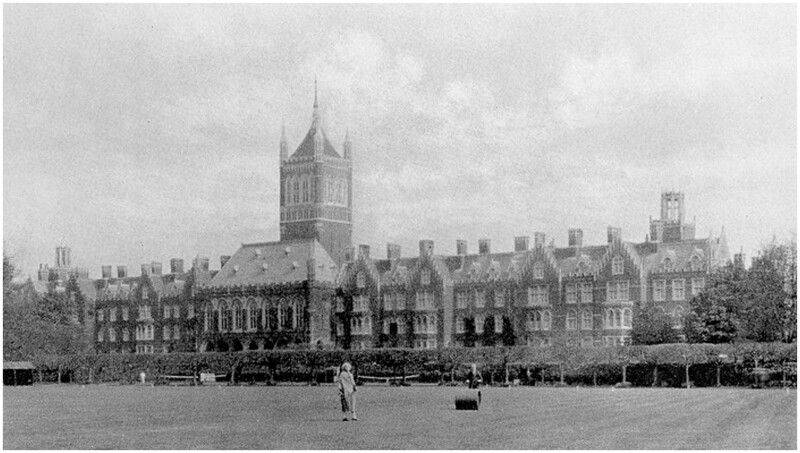
Holloway Sanatorium, Virginia Water, 1885 (Wellcome Images).

No longer occupied with medical work, Barker’s health deteriorated rapidly. On 23 March 1896, she was taken to the Holloway Sanatorium at Virginia Water by her brother, Frederick (Figure 3).^117^ The occupation which had defined her life for more than a decade—‘Doctor of Medicine’—was recorded on admission alongside the diagnosis which would remain with her for the next 50 years: ‘Chronic Mania’.^118^ The underlying organic cause of Barker’s mania, as defined by the medical officer who admitted her, is unknown. No family history of mental illness is recorded in Barker’s case notes; however, research has revealed that her younger sister, Emma, similarly died in a mental institution in 1938, aged 81 years.^119^ In a cruel twist of fate, Barker’s name and qualifications continued to be listed on the British Medical Register until 1903.^120^ Similarly, her details were reprinted in *The Medical Directory* until 1905, 9 years after becoming a patient in Holloway, with the final entry noting that her address was ‘uncommunicated’.^121^ Barker’s arrival in Virginia Water marked the end of her life of responsibility and purpose as a practising physician. This being said, the 50 years that she spent as a patient is no less important. Surviving case books offer fascinating insight into how Barker’s former profession continued to influence her reality within the walls of the sanatorium. Her unwavering refusal to show any sign of weakness, decades after she first became a patient, further supports the view that the pressures faced by these early women doctors had devastating and long-lasting effects on Barker.

## Holloway Sanatorium

Founded in 1885 by businessman and philanthropist Thomas Holloway, Holloway Sanatorium was originally intended as a refuge for those of the middle classes who had been ‘temporarily deprived of their reason’, offering care ‘at charges suited to their means’.^122^ Set in 22 acres of park land, the Sanatorium boasted ‘opulent … front rooms and suites’ for those who could afford it, and provided ‘apartments in less prominent parts of the building’ for those less fortunate.^123^ Being situated less than 30 miles from Aldershot, Virginia Water would have been the obvious choice for the Barker family. Unfortunately, the case book which contains Barker’s admission notes, a photograph and a further 11 years of medical records dating from 1896 to 1907, has not survived the passing of time.^124^ In their absence, what can be deduced is that either her illness had worsened to the extent that she could no longer be cared for by her family at home, or her family’s circumstances had changed. Interestingly, it is noted in the admission register that Barker’s ‘Age on first Attack’ was 30, which would coincide with the beginning of her decline whilst working at the BMHW in the early 1880s.

The first surviving entry which refers to Barker dates from 11 July 1907, 11 years after her admission to Holloway:

Patient continues in a state of chronic mania asserting that she has sovereign right here, always asking for a cab to drive to Buckingham Palace or Aldershot. Jealous of any authority other than her own, forbidding the doctors to go near patients etc etc—She is occasionally noisy at night—She refuses any physical examination.^125^

Barker’s memories of her past evidently remained at the forefront of her mind throughout her time spent at Virginia Water. Her delusions of grandeur, viewed as being indicative of her ‘Chronic Mania’, were inextricably intertwined with her lived experiences as a medical practitioner. Barker’s refusal to accept the authority of her doctors, and her refusal to be examined or show any sign of physical weakness suggests that she could not accept, or understand, the passive role of patient in which she now found herself. Barker’s attempts to go home to Aldershot, a place of familiarity intimately connected with her past, similarly conveys that in her distorted version of reality, everything remained as it had been; she was a doctor, and her work was not yet done.

Barker’s staunch resistance to medical authority did not abate; despite having not practised medicine in a hospital setting for more than a decade, she continued to be drawn to the ‘tools’ of her trade, with stethoscopes and keys serving as tangible reminders of the responsibility she once held:

‘5^th^ January 1909 – Patient … will have nothing to do with the A.M.O, stating that she is the only Dr here’.

‘2^nd^ April 1909 – Continues mildly excited, asking that keys stethoscope etc may be given to her, as she has sovereign right here’.

‘10^th^ January 1910 – Thinks she is the only Doctor in the place, which belongs to her. Appears in good physical health’.

‘19^th^ July 1915 – Demented, has grandiose delusions – says that she is the Queen of England and frequently calls for imaginary policemen to arrest the nurses and M.O … has no useful occupation’.

‘9^th^ February 1920 – Still calls herself a Queen, also that she is the only doctor here, tried to snatch away my stethoscope saying it was hers’.^126^

Much to the dismay of the Sanatorium’s Medical Officers, Barker continued to refuse all physical examinations, which meant that the staff had to presume, from outward appearances alone, that she was free from illness and disease: ‘She *appears* in good physical health, except for an occasional cold for which she *always* refuses treatment’.^127^

In 1921, a quarter century after entering the Sanatorium, Barker was forced to show weakness and accept help from those who were caring for her. At 70 years, she fell and broke her leg, leaving her with no option but to recognise that she was not, in fact, ‘the only doctor in this place’. In an uncharacteristic, yet touching, mark of deference, the Medical Officer (MO) used Barker’s professional title in their account of her accident: ‘Dr Barker slipped in the gallery today and fell on her left side, fracturing the neck of her left femur. *She objected much to being nursed but in the end allowed herself to be undressed and X-rayed*’.^128^ The reason why the MO chose to use Barker’s title in this particular moment is open to interpretation; perhaps they were taken aback by her unwavering resolve, moved by the extent to which she would try to cling onto her independence, in spite of excruciating pain. In the following entry, written in the same hand 2 months later, Barker is once again referred to as the ‘patient’, the glimmer of respect for her having passed: ‘Patient is now able to stand on her leg and walk a little. *She seems to get a considerable amount of pain but will not allow examination* … She is looking thinner and paler than before the accident.’^129^ A year after her fall, Barker was noted to have made a satisfactory physical recovery but her ‘exulted delusions’ remained unchanged.^130^

Perhaps unsurprisingly, given her unwavering resolve, Barker’s last surviving entry in the patient case book, written three decades after her admission to Virginia Water, echoes her first. Barker’s past life as a doctor remains firmly present in the foreground of her confused reality: ‘Asks almost every day for her “medical and surgical things” and always wants my stethoscope when she sees it.’^131^ On 2 June 1945, aged 93, Barker passed away having spent the majority of her life as a patient in Holloway Sanatorium.^132^ Her estate, worth in excess of £50,000, remained unclaimed until 1949, when a distant relative was eventually found.^133^ In spite of remaining on the periphery of her professional circle throughout her career, as one of its youngest members, Barker went on to outlive all of her former colleagues. Although she had ceased treating patients by the late 1880s, in her own mind at least, she continued to ‘practice’ medicine well into her old age. Given that the details of Barker’s former life no longer existed in living memory, she received no obituary in the medical or lay press.^134^

By shedding new light on Barker’s story, and the internal dynamics of the professional group of which she was a part, much can be learnt about the numerous pressures faced by early medical women. Once qualified, women doctors were expected to act as part of their new community, rather than as individuals. As Garrett Anderson remarks in her inaugural address at the LSMW in 1877, ‘the future success of our cause depends very much upon the judgement and moderation, as well as upon the zeal of its earliest advocates … *you are not mere isolated units in society’*.^135^ There was an acute expectation for women doctors to uphold the ‘zeal’ which the movement was founded upon. Professional disagreements were commonplace, in spite of Garrett Anderson’s advice that medical women should ‘free [themselves] from petty jealousies … promote the highest aims and interests of the profession, to purge it of its flaws and to add to its honour’.^136^ Hospital practice was similarly fraught for women doctors, who were faced with the task of upholding the ‘honour’ of all medical women whilst working alongside their male colleagues in an unforgiving environment. For some, this great responsibility was evidently insurmountable. As one of the youngest members of the first generation of women doctors, Barker’s committal provides fascinating insight into the enduring effects of this period of conflict on those fighting on the frontline. Her story demonstrates that, for many women doctors, the victories won at the end of the nineteenth century came at a remarkably high price.

## Footnotes

1 Examples include Sophia Jex-Blake, *Medical Women: A Thesis and a History* (Edinburgh: Oliphant & Co., 1886), E. Moberley Bell, *Storming the Citadel* (London: Constable & Co, 1982), and Catriona Blake, *The Charge of the Parasols* (London: The Women’s Press, 1990).

2 Barker is briefly mentioned in: M. Anne Crowther and Marguerite W. Dupree, *Medical Lives in the Age of Surgical Revolution* (Cambridge: Cambridge University Press, 2007) and Judith Lockhart, ‘Women, Health and Hospitals in Birmingham: The Birmingham and Midland Hospital for Women’ (unpublished doctoral thesis, University of Warwick, 2008).

3 Elizabeth Garrett Anderson, ‘A Special Chapter for Ladies Who Propose to Study Medicine’, in Charles Bell Keetley, ed., *The Medical Student’s Guide to the Medical Profession* (London: Macmillan and Co., 1878), 42–48, 43.

4 Virginia Drachman, ‘Female Solidarity and Professional Success: The Dilemma of Women Doctors in Nineteenth-Century America’, *Journal of Social History*, 1981–82), 15, 607–19, 607. [Emphasis added.]

5 Jo Manton, *Elizabeth Garrett Anderson* (London: Butler and Tanner, 1965), 147.

6 Elizabeth Blackwell*, Pioneer Work in Opening the Medical Profession to Women* (New York: Longmans, Green & Co., 1895), 184.

7 *Ibid*., 163. For more on Garrett Anderson’s fight for a medical education, see Louisa Garett Anderson, *Elizabeth Garrett Anderson, 1836–1917* (London: Faber & Faber, 1939).

8 *Ibid*. Elizabeth Blackwell (1821–1910) was the first woman to have her name placed on the British Medical Register in 1858, in spite of having a foreign medical degree. This was because she met the two requirements of the Medical Act of 1858: her degree had been awarded before 1858, and she could also prove that she had practised medicine in England before this time.

9 Margaret Todd, *The Life of Sophia Jex-Blake* (London: Macmillan, 1918), 232.

10 Jex-Blake, *Medical Women*, 70.

11 ‘Medical Act 1858’ <www.legislation.gov.uk> [accessed 24 February 2018].

12 Manton, *Elizabeth Garrett Anderson*, 122.

13 For a contemporary reaction to Jex-Blake’s actions, see Frances Hoggan, ‘Women in Medicine’, in Theodore Stanton, ed., *The Woman Question in Europe* (New York: G.P. Putnam's Sons, 1884), 63–90.

14 ‘Annie Reay Barker’, England and Wales Civil Registration Birth Index, <www.ancestry.com> [accessed 6 November 2018].

15 Anon., ‘Edmund John Barker, M.D, M.R.C.S, L.S.A’, *British Medical Journal*, 1888, 2, 396.

16 J. W. Byers, ‘Introductory Address of the London (Royal Free Hospital) School of Medicine for Women’, *The Lancet*, 1906, 168, 927–29, 927.

17 For more on Byers, see Alison Jordan, *Margaret Byers, Pioneer of Women’s Education and Founder of Victoria College, Belfast* (Belfast: Queen’s University of Belfast, 1991).

18 Sophia Jex-Blake, ‘Medical Education for Women’, *The Times*, 28 July 1869, 10.

19 First Matriculation Album 1869–1870, Centre for Research Collections, University of Edinburgh, EUA IN1/ADS/STA/1. Helen Grant signed the register in her maiden name, as was the Scottish custom. She was a widow, and is often referred to by her married name—Helen de Lacey Evans.

20 First Matriculation Album 1870–1871, EUA/IN1/ADS/STA/2.

21 *Ibid*., 39.

22 *Ibid*., 39

23 First Matriculation Album 1869–1870, EUA IN1/ADS/STA/1. Death dates have proved impossible to trace for these women, as they did not go on to become medical practitioners. Masson (née Orme) was a prominent suffrage campaigner in Scotland, and was leader of the Edinburgh National Society for Women’s Suffrage.

24 Jex-Blake, *Medical Women*, preface.

25 *Ibid*.

26 *Ibid*., 41–42.

27 Crowther and Dupree, *Medical Lives*, 39.

28 Todd, *The Life of Sophia Jex-Blake*, 278.

29 Crowther and Dupree, *Medical Lives*, 42.

30 Jas Dingwall Fordyce *et al.*, *The Scottish Law Reporter, Volume X, October 1872*–*October 1873* (Edinburgh: John Baxter, 1873), 553. Russell Rorison completed her medical degree at the London School of Medicine for Women, graduating in 1880.

31 *Ibid*. None of these women went on to become medical practitioners, hence their birth and death dates have proved impossible to trace. Shedlock continued her medical studies in Paris between 1873 and 1875, but died in Madeira in 1878. ‘Student of Medicine’ was recorded on her death certificate. See Neil McIntyre, ‘The Fate of Rose Anna Shedlock (c1850–1878) and the Early Career of Émile Roux (1853–1933)’, *Journal of Medical Biography*, 2014, 24, 60–67.

32 Elizabeth Garrett Anderson, ‘The Medical Education of Women’, *The Times*, 5 August 1873, 3; Sophia Jex-Blake, ‘The Medical Education of Women’, *The Times*, 23 August 1873, 4. [Emphasis added.] Garrett Anderson received her M.D. from the Universitié de Paris in 1870.

33 For more on Dahms, see Peter Mohr, ‘Women-run Hospitals in Britain: A Historical Survey a Focusing on Dr Catherine Chisholm (1878-1952) and The Manchester Babies' Hospital’ (unpublished doctoral thesis, University of Manchester, 1995).

34 Minute Book of the Medical Committee, HC/WH/5/1, Birmingham City Archives, Library of Birmingham, 16 July 1872.

35 ‘Hospital and School News’, *London (Royal Free Hospital) School of Medicine for Women Magazine*, 19 May 1901, 800. The LSMW became affiliated with the Royal Free Hospital in 1877.

36 BMHW Annual Report 1873, HC/WH/1/10/1, BCA.

37 Minute Book of the Medical Committee, 31 July 1875.

38 Board of Governors Minutes, HCIWH1/1/1, 31 July 1875, BCA.

39 Lockhart, ‘Women, Health and Hospitals in Birmingham’, 74.

40 *Ibid*., 46.

41 *Ibid*., 52.

42 *Ibid*.

43 'Medical Vacancies', *British Medical Journal*, 1876, 1, 743.

44 Mary Ann Elston, ‘Women Doctors in the British Health Services, a Sociological Study of Their Careers and Opportunities’ (unpublished doctoral thesis, University of Leeds, 1986), 224.

45 Salaries advertised in the *BMJ* for House Surgeon positions ranged from £50 to £120 in June 1876. Barker’s salary was comparable to that offered to Medical Officers in poor law unions, which typically ranged from £40 to £95 per annum. Union medical officers were required to be fully licensed in surgery.

46 Board of Governors Minutes, 6 July 1876.

47 BMHW Annual Report 1876.

48 Barker’s thesis translates as: ‘Considerations on Caring for Women, Without any Accident, Before, During, and after Labour’.

49 Anon., *The Law Reports* (London: William Clowes and Sons, 1876), 289.

50 Laura Kelly, *Irish Women in Medicine, C.1880’s-1920’s: Origins, Education and Careers* (Manchester: Manchester University Press, 2012), 39–40.

51 *Ibid*., 9.

52 *Ibid*., 11.

53 Letter from Frederick Rowland Barker to his parents, 18 December 1877, Royal Army Medical Corps Archive, Wellcome Library, London, (RAMC/347/4).

54 Kelly, *Irish Medical Women*, 43. It is possible that the ‘failure’ refers to the snub which Jex-Blake encountered when she was overlooked for the position of secretary at the LSMW in June 1877.

55 Todd, *The Life of Sophia Jex-Blake*, 439. For more on the issues surrounding Todd’s biography of Jex-Blake, see Kristine Swenson, ‘Intimate Sympathy and Self-Effacement: Writing the Life of Sophia Jex-Blake’, *a/b: Auto/Biography Studies*, 2014, 14, 222–40.

56 *Ibid*., 438.

57 Roll of Licentiates in Medicine and Midwifery 1866–1948, Royal College of Physicians of Ireland Heritage Centre, RCPI/5/2/1/3.

58 Board of Governors Minutes, 2 April 1878.

59 ‘Birmingham and Midland Hospital for Women’, *Birmingham Daily Post*, 7 June 1878, 1.

60 Claire Brock. *British Women Surgeons and their Patients, 1860*–*1918* (Cambridge: Cambridge University Press, 2017), 19.

61 Minute Book of the Medical Committee, 11 July 1878.

62 Lockhart, ‘Women, Health and Hospitals in Birmingham’, 75.

63 Board of Governors Minutes, no date recorded, 190.

64 *The Medical Examiner, Volume III, January to June 1878*, ed. Oakley Coles (London: C.W. Reynell, 1878), 56.

65 ‘Address by Mr Lawson Tait’, *The British Gynaecological Journal*, 1886, 2, 19–29, 22.

66 *Ibid*., 22–23. [Emphasis added.]

67 Elizabeth Garrett Anderson, *Inaugural Address* (London: H.K Lewis, 1877), 20–21. [Emphasis added.]

68 ‘Address by Mr Lawson Tait’, 23–24.

69 Ben Griffen, *The Politics of Gender in Victorian Britain, Masculinity, Political Culture and the Struggle for Women's Rights* (Cambridge: Cambridge University Press, 2012), 310.

70 *Ibid*., 184.

71 *Ibid*., 24. [Emphasis added.] For more on surgical risk and controversy in the late nineteenth century, see Claire Brock, *British Women Surgeons and their Patients, 1860*–*1918* (Cambridge: Cambridge University Press, 2017).

72 Sally Frampton, *Belly-Rippers, Surgical Innovation and the Ovariotomy Controversy* (Switzerland: Palgrave Macmillan, 2018), 120.

73 *Ibid*., 147.

74 Tara Lamont, ‘The Amazons Within: Women in the BMA 100 Years Ago’, *British Medical Journal*, 1992, 305, 1529–32, 1531.

75 *Ibid*., 1530.

76 *Ibid*.

77 Association of Registered Medical Women Minute Book, Wellcome Library, London, 6 May 1879, SA/MWF/C.74.

78 *Ibid*., 4 May 1880.

79 *Ibid*.

80 Mary Marshall, Julia Cock (1860–1914), Florence Nightingale Boyd (1867–1910) and May Thorne (1861–1951) were also elected as president three times, whilst the future president of the Medical Women’s Federation (MWF), Jane Walker (1859–1938), was elected four times.

81 Ellen S. More, ‘The Blackwell Medical Society and the Professionalization of Women Physicians’, *Bulletin of the History of Medicine*, 1987, 61, 603–28, 603.

82 Minutes of the Annual Meeting, 12 June 1883.

83 Michaelson, ‘Becoming “Medical Women”’, 123. [Emphasis added.]

84 Whilst the official constitution of the ARMW is referred to as early as 1882, the first printed copy dates from 1892.

85 Minutes of the Annual Meeting, 4 June 1884.

86 *Ibid*.

87 Todd, *The Life of Sophia Jex-Blake*, 279

88 Jex-Blake, *Medical Women*, 156–7.

89 Jo Manton, *Elizabeth Garrett Anderson* (London: Butler and Tanner, 1965), 212.

90 *Ibid*., 228.

91 *Ibid*.

92 *Ibid*., 240.

93 *The Englishwoman’s Review of Social and Industrial Questions, Volume XII, January to December 1881*, ed. C. A. Biggs (London: Englishwoman’s Review, 1881), 466–67.

94 *Ibid*.

95 Minute Book of the Medical Committee.

96 Board of Governors Minutes, 3 October 1882.

97 *Ibid*., 7 November 1882.

98 *Ibid*., 6 February 1883.

99 *The Englishwoman’s Review of Social and Industrial Questions, Volume XVI, January to December 1885*, ed. C.A. Biggs (London: Englishwoman’s Review, 1885), 66; *The Englishwoman’s Review of Social and Industrial Questions, Volume XVIII, January to December 1887*, ed. C. A. Biggs (London: Englishwoman’s Review, 1887), 69.

100 ‘Louisa Atkins, M.D’, *British Medical Journal*, 1924, 2, 836–37, 836.

101 Minutes of the Annual Meeting, 11 June 1889.

102 Brock, *British Women Surgeons*, 37.

103 Minutes of the Annual Meeting, 14 June 1887.

104 Minutes of the Annual Meeting, 12 June 1888.

105 Elston, ‘Women Doctors in the British Health Services’,156.

106 ‘Louisa Atkins, M.D’, 836–37. It is interesting to note that neither Barker or Atkins have any surviving photographs, further evidence that both eschewed all forms of publicity throughout their lives.

107 Clare Gerada, ‘Doctors and Suicide’, *British Journal of General Practice*, 2018, 68, 168–69, 168.

108 *Ibid*.

109 Claire Brock, ‘The Disappearance of Sophia Frances Hickman, M.D.’, *History Workshop Journal*, 2015, 80, 161–82, 161.

110 *Ibid*., 177.

111 *Ibid*., 178.

112 *The Englishwoman’s Review of Social and Industrial Questions, Volume XIX, January to December 1888*, ed. C.A. Biggs (London: Englishwoman’s Review, 1888), 59.

113 London School of Medicine for Women Annual Reports, London Metropolitan Archives, H72/SM/A/O1/01/002.

114 See Andrew Scull (ed.), *Madhouses, Mad-Doctors, and Madmen: The Social History of Psychiatry in the Victorian Era* (Pennsylvania: University of Pennsylvania Press, 2017).

115 Brock, ‘The Disappearance of Sophia Frances Hickman, M.D.’, 161.

116 *Ibid*.

117 Holloway Sanatorium Patient Admission Register, Surrey History Centre, Patient number 1591, 3237/5/1.

118 For more on (male) medical practitioner’s experiences as patients in Asylums, see Allanah Tomkins, ‘Mad Doctors? The Significance of Medical Practitioners Admitted as Patients to the first English County Asylums up to 1890’, *History of Psychiatry*, 2012, 4, 437–53.

119 ‘Emma Florence Coburn’, UK Newspaper Index, <www.ancestry.com> [accessed 6 November 2019]. The extent of Emma’s illness and the exact date of her admission to Brooke House Mental Hospital are unknown. After marrying in 1898, she disappears from the address shared with her husband in the 1901 census in 1911, suggesting that her admission is likely to have occurred during this decade.

120 ‘Annie Barker’, UK Medical Registers 1859–1959, <www.ancestry.com> [accessed 16 November 2018].

121 ‘Annie Barker’, UK & Ireland Medical Directories 1845–1942, <www.ancestry.com> [accessed 9 November 2018].

122 ‘Holloway Sanatorium’, *Surrey Advertiser*, 20 June 1855, 5.

123 *Ibid*. For more on Holloway Sanatorium, see Anna Shephard, *Institutionalizing the Insane in Nineteenth-Century England* (Routledge: Oxon, 2014).

124 Supplementary Patient Case Book, February 1887–May 1926, Surrey History Centre, Surrey, 3473/3/1/1/18. The first case book entry in July 1907 refers to a continuation from ‘Book IX 135-178’.

125 Patient Case Book, 3473/3/1/1/18, SHC.

126 *Ibid*.

127 *Ibid*., 20 October 1910. [Emphasis added.]

128 *Ibid*., 27 June 1921. [Emphasis added.]

129 *Ibid*., 22 August 1921. [Emphasis added.]

130 *Ibid*., 29 August 1922.

131 *Ibid*., 1 April 1926. Due to an imposed closure period, Barker’s records from 1926 to 1945 are unavailable.

132 In spite of extensive research, Barker’s final resting place has not been found. It is likely that, given she had no surviving family, her funeral was organised by the Parish, and she was buried in an unmarked grave.

133 ‘Annie Reay Barker’, English Newspaper Index Cards, 1790–1976, <www.ancestry.com> [accessed 16 November 2018].

134 It was common place for women doctors active in the late nineteenth century to have obituaries featured in the Medical Women’s Federation *Newsletter* and *Journal*.

135 Garrett Anderson, *Inaugural Address*, 20–21. [Emphasis added.]

136 *Ibid*.

